# Trypanosoma cruzi Cardiomyopathy Diagnosed via Explant Tissue 28S Ribosomal Gene Sequencing Following Orthotopic Heart Transplantation

**DOI:** 10.7759/cureus.112969

**Published:** 2026-07-19

**Authors:** Aneurin C Fernandez, Gerald J Berry, Ragini Ahanonu, Jessica Ferguson Toll

**Affiliations:** 1 Division of Infectious Diseases and Geographic Medicine, Stanford University, Stanford, USA; 2 Department of Pathology, Stanford University School of Medicine, Stanford, USA; 3 Department of Pathology, Stanford University, Stanford, USA

**Keywords:** 28s sequencing, chagas cardiomyopathy, immunosuppression therapy, orthotopic heart transplant, trypanosoma cruzi

## Abstract

*Trypanosoma cruzi*, the protozoal agent responsible for Chagas disease, is a well-established cause of infectious cardiomyopathy. Diagnosing chronic Chagas cardiomyopathy can be exceptionally challenging due to the sparse presence of amastigotes in explanted cardiac tissue. We present a case of a 53-year-old male originally from Veracruz, Mexico, residing in the United States, who presented with rapidly progressive biventricular heart failure refractory to goal-directed medical therapy. The patient developed cardiogenic shock necessitating mechanical circulatory support and subsequent orthotopic heart transplantation. Histopathological evaluation of the explanted heart revealed end-stage dilated cardiomyopathy with mixed inflammatory infiltrates and poorly formed granulomas, but no visible amastigotes. Given the granulomatous pathology, a lab-developed 28S ribosomal gene sequencing assay was performed on the myocardial tissue, successfully identifying *Trypanosoma cruzi*. Subsequent recipient serological testing confirmed the diagnosis. Post-transplant molecular surveillance remained negative for parasitic reactivation under immunosuppression. This case highlights the critical utility of advanced molecular diagnostic techniques, such as tissue-based ribosomal gene sequencing, alongside traditional dual-methodology serology, to establish a definitive diagnosis of Chagas disease in atypical presentations and ensure appropriate post-transplant monitoring.

## Introduction

*Trypanosoma cruzi,* a protozoal parasite transmitted primarily by triatomine insects, is the causative agent of Chagas disease [1]. Up to 30% of chronically infected individuals eventually develop chronic Chagas cardiomyopathy, often culminating in refractory biventricular heart failure, life-threatening ventricular arrhythmias, and thromboembolic events [2].

When patients progress to end-stage heart failure, orthotopic heart transplantation (OHT) is often the only remaining definitive intervention. However, managing Chagas disease in the transplant setting presents unique clinical challenges. Confirming the diagnosis from explanted myocardial tissue is notoriously difficult; chronic Chagas cardiomyopathy is characterized by intense fibrotic remodeling and low-grade mononuclear inflammation, meaning visible *T. cruzi *amastigotes are exceptionally sparse or absent [3]. Post-transplant immunosuppressive regimens significantly elevate the risk of parasitic reactivation, which can manifest as acute myocarditis or cutaneous lesions, and demand vigilant postoperative monitoring [2].

Traditional diagnosis relies on dual-methodology serology, but advanced molecular techniques have fundamentally changed how we identify atypical presentations. Targeted ribosomal gene sequencing could enable the identification of pathogen DNA directly from formalin-fixed, paraffin-embedded (FFPE) tissue blocks even when conventional histopathology is negative. In this report, we present a rare case of *T. cruzi* cardiomyopathy diagnosed via explant tissue 28S ribosomal gene sequencing (a broad-range molecular assay used to detect fungal and protozoal pathogens) following OHT, emphasizing the utility of molecular diagnostics in modern transplant medicine.

## Case presentation

**Figure 1:**
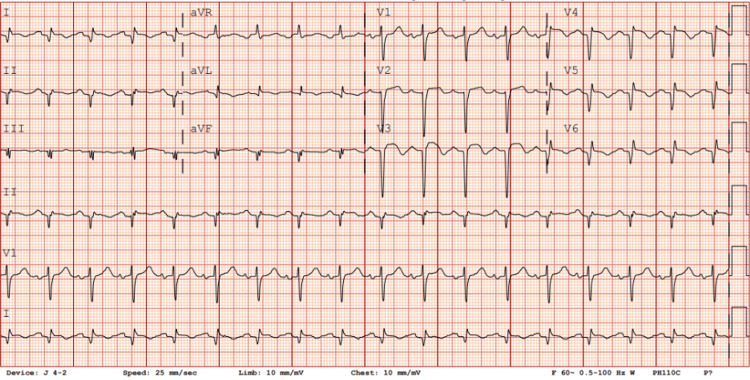
Twelve-lead electrocardiogram (EKG) demonstrating low-voltage QRS complexes in the limb leads and left axis deviation.

A 53-year-old male from a rural area of Veracruz, Mexico, with a history of dyslipidemia, presented with sudden-onset chest pain and shortness of breath while working in construction. An electrocardiogram revealed low-voltage QRS complexes and left axis deviation (Figure 1). Brain natriuretic peptide levels were elevated at 1,785 pg/ml (reference <100 pg/ml). A transthoracic echocardiogram demonstrated severely dilated bilateral ventricles with a significantly reduced left ventricular ejection fraction of 15-20%, indicating severe global hypokinesis. Coronary angiography revealed mild non-obstructive coronary artery disease. A urine toxicology screen was negative. The etiology of cardiomyopathy remained unclear.

**Figure 2:**
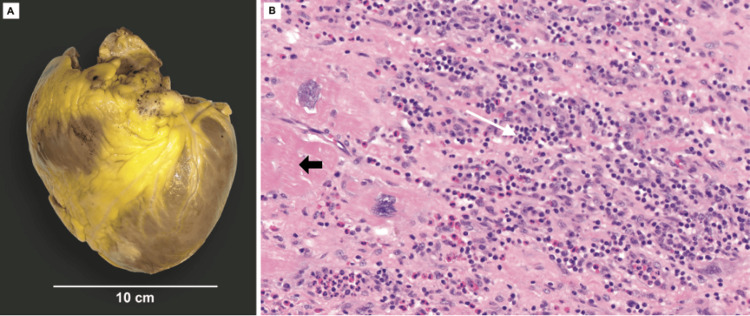
(A) Gross pathological examination of the explanted heart demonstrates end-stage dilated cardiomyopathy with marked thinning of the ventricular walls and global enlargement. (B) Histopathological section (hematoxylin and eosin stain, original magnification x200) showing dense interstitial fibrosis (black arrow) and chronic inflammatory infiltrate (white arrow).

Despite starting goal-directed medical therapy, his condition progressed, and he was found to be in cardiogenic shock. This required placement of an intra-aortic balloon pump and eventually an orthotopic heart transplant 24 days after his initial presentation. Gross examination of the explanted heart showed cardiomegaly with biventricular hypertrophy and dilatation (Figure 2A). Histological examination revealed end-stage dilated cardiomyopathy, characterized by myocyte hypertrophy and interstitial fibrosis. It also had mixed inflammatory cell infiltrates - lymphocytes, histiocytes, eosinophils, and poorly formed granulomas (Figure 2B).

While amastigotes are rarely seen in chronic stages of Chagas cardiomyopathy, the presence of granulomatous inflammation prompted further investigation [1]. A lab-developed 28S ribosomal gene sequencing assay of heart tissue identified *Trypanosoma cruzi*. Likely due to the extreme clinical acuity of the patient’s presenting cardiogenic shock, only standard pre-transplantation screening serologies were obtained, and targeted epidemiologic-risk screening, including *T. cruzi *serology, was deferred. Subsequent recipient *T. cruzi* serological testing post-transplant was positive via enzyme-linked immunosorbent assay (ELISA) and lateral flow assay (LFA). In line with recommended protocols, post-transplant monitoring with serial polymerase chain reaction (PCR) by the Centers for Disease Control and Prevention (CDC) was performed weekly for the first two months post-transplant, biweekly during the third month, and monthly thereafter for at least six months, and remained negative [4,5].

## Discussion

*Trypanosoma cruzi*, the protozoal agent of Chagas disease, is endemic to Mexico, Central America, and South America [6]. Recent medical literature has reported an increasing number of autochthonous cases in the United States [7]. Transmission occurs through triatomine bug bites and feces, blood transfusions, vertical transmission, or consumption of contaminated food and water [8]. The infection progresses through an acute phase, which is often asymptomatic, followed by a chronic phase. Symptomatic acute phase may present with lesions at the inoculation site (e.g., chagoma or Romaña sign), fever, fatigue, lymphadenopathy, and hepatosplenomegaly. This phase typically self-resolves within four to eight weeks [6]. If left untreated, the condition can transition to an indeterminate form (the majority) or a chronic phase marked by visceral involvement. Cardiac Chagas, the most common chronic manifestation, entails electrophysiological abnormalities and dilated cardiomyopathy; whereas gastrointestinal Chagas can lead to megaesophagus and/or megacolon [6].

Diagnosis shifts from detection of trypomastigotes from body fluids in the acute phase to serology in the chronic phase. Two serological tests with different methodologies are recommended, with a third method to be utilized as a tiebreaker [4].

In solid organ transplantation, the risk of transmission is highest with heart transplants from infected donors (~75%). For transplants involving seropositive donors or recipients, PCR surveillance is critical to detect reactivation. Molecular surveillance is recommended weekly for the first two months post-transplant, biweekly during the third month, and monthly thereafter for at least six months, with the total duration of testing tailored to the recipient's net state of immunosuppression [5]. This strategy is designed to detect low-level parasitemia weeks before clinical symptoms or histological evidence of tissue reactivation manifest, allowing for preemptive therapy. While benznidazole or nifurtimox are used to treat reactivation, their use as prophylaxis is limited by toxicity and interactions with immunosuppressants like calcineurin inhibitors. Reactivation may manifest as fevers, skin lesions, myocarditis, and meningoencephalitis [9].

Diagnosing an unrecognized *T. cruzi *cardiomyopathy from explant tissue presents a classic diagnostic challenge. While post-transplant protocols heavily rely on prospective blood PCR surveillance to catch early parasitemia [5], identifying chronic Chagasic changes in the explanted heart itself is often limited by traditional histopathology. Standard H&E staining frequently misses the diagnosis because parasite distribution is highly focal, and classic amastigote nests are rarely conspicuous during chronic stages [10]. Other molecular strategies, such as multi-target real-time PCR assays on blood and tissue specimens, have been utilized to improve sensitivity [11]; however, our case highlights how 28S ribosomal gene sequencing on explant tissue can successfully bridge this diagnostic gap. Ultimately, maintaining a high index of suspicion in patients from endemic areas is critical; by relying on rapid, highly specific molecular sequencing when morphology or serology is inconclusive, clinical teams can immediately initiate postoperative PCR monitoring to prevent systemic reactivation [11].

A key limitation of this diagnostic pathway is that post-transplant explant tissue sequencing is a retroactive measure and does not replace standard, epidemiologically guided pre-transplant screening. In elective settings, preoperative serological screening triggers proactive, preventive clinical management. However, in emergent transplant scenarios, post-transplant tissue molecular diagnostics could serve as an essential safety net to guide subsequent monitoring rather than a substitute for standard preoperative protocols.

## Conclusions

This case demonstrates that chronic Chagas cardiomyopathy can present as a rapidly progressive biventricular heart failure requiring emergency OHT. Confirming Trypanosoma cruzi infection on explanted cardiac tissue remains a notorious diagnostic challenge due to the lack of visible amastigotes in the chronic phase. Utilizing advanced molecular diagnostics, such as 28S ribosomal gene sequencing on paraffin-embedded tissue blocks, offers a useful adjunctive tool to secure a definitive diagnosis when traditional pathology is unrevealing. Ultimately, combining tissue-based molecular assays with dual-methodology serology is essential to establish the correct diagnosis early, enabling clinicians to initiate appropriate post-transplant PCR surveillance and safely manage the risk of parasitic reactivation under intense immunosuppression.

## References

[1] Kransdorf EP, Fishbein MC, Czer LS (2016). Pathology of chronic Chagas cardiomyopathy in the United States: a detailed review of 13 cardiectomy cases. Am J Clin Pathol.

[2] Pinazo MJ, Miranda B, Rodríguez-Villar C (2011). Recommendations for management of Chagas disease in organ and hematopoietic tissue transplantation programs in nonendemic areas. Transplant Rev (Orlando).

[3] Bonney KM, Luthringer DJ, Kim SA, Garg NJ, Engman DM (2019). Pathology and pathogenesis of Chagas heart disease. Annu Rev Pathol.

[4] (2026). CDC. American Trypanosomiasis. http:////www.cdc.gov/dpdx/trypanosomiasisamerican/index.html.

[5] La Hoz RM, Morris MI (2019). Tissue and blood protozoa including toxoplasmosis, Chagas disease, leishmaniasis, Babesia, Acanthamoeba, Balamuthia, and Naegleria in solid organ transplant recipients— guidelines from the American Society of Transplantation Infectious Diseases Community of Practice. Clin Transplant.

[6] Pérez-Molina JA, Molina I (2018). Chagas disease. Lancet.

[7] Beatty NL, Hamer GL, Moreno-Peniche B, Mayes B, Hamer SA (2025). Chagas disease, an endemic disease in the United States. Emerg Infect Dis.

[8] Swett MC, Rayes DL, Campos SV, Kumar RN (2024). Chagas disease: epidemiology, diagnosis, and treatment. Curr Cardiol Rep.

[9] Pierrotti LC, Carvalho NB, Amorin JP, Pascual J, Kotton CN, López-Vélez R (2018). Chagas disease recommendations for solid-organ transplant recipients and donors. Transplantation.

[10] Azevedo PH, Xavier MA, Silva GN, Costa PA, Carneiro CM, Brasileiro Filho G (2018). Anti-serum validation for use in immunohistochemistry for Trypanosoma cruzi detection. Rev Soc Bras Med Trop.

[11] Qvarnstrom Y, Schijman AG, Veron V, Aznar C, Steurer F, da Silva AJ (2012). Sensitive and specific detection of Trypanosoma cruzi DNA in clinical specimens using a multi-target real-time PCR approach. PLoS Negl Trop Dis.

